# Analysis of aptamer-target binding and molecular mechanisms by thermofluorimetric analysis and molecular dynamics simulation

**DOI:** 10.3389/fchem.2023.1144347

**Published:** 2023-05-09

**Authors:** Hong-Li Zhang, Cong Lv, Zi-Hua Li, Song Jiang, Dan Cai, Shao-Song Liu, Ting Wang, Kun-He Zhang

**Affiliations:** Department of Gastroenterology, The First Affiliated Hospital of Nanchang University, Jiangxi Institute of Gastroenterology and Hepatology, Jiangxi Clinical Research Center for Gastroenterology, Nanchang, China

**Keywords:** aptamers, thermofluorimetric analysis, molecular dynamics simulation, reaction condition optimization, aptamer selection

## Abstract

**Introduction:** Aptamers are valuable for bioassays, but aptamer-target binding is susceptible to reaction conditions. In this study, we combined thermofluorimetric analysis (TFA) and molecular dynamics (MD) simulations to optimize aptamer-target binding, explore underlying mechanisms and select preferred aptamer.

**Methods:** Alpha-fetoprotein (AFP) aptamer AP273 (as the model) was incubated with AFP under various experimental conditions, and melting curves were measured in a real-time PCR system to select the optimal binding conditions. The intermolecular interactions of AP273-AFP were analysed by MD simulations with these conditions to reveal the underlying mechanisms. A comparative study between AP273 and control aptamer AP-L3-4 was performed to validate the value of combined TFA and MD simulation in selecting preferred aptamers.

**Results:** The optimal aptamer concentration and buffer system were easily determined from the dF/dT peak characteristics and the melting temperature (Tm) values on the melting curves of related TFA experiments, respectively. A high Tm value was found in TFA experiments performed in buffer systems with low metal ion strength. The molecular docking and MD simulation analyses revealed the underlying mechanisms of the TFA results, i.e., the binding force and stability of AP273 to AFP were affected by the number of binding sites, frequency and distance of hydrogen bonds, and binding free energies; these factors varied in different buffer and metal ion conditions. The comparative study showed that AP273 was superior to the homologous aptamer AP-L3-4.

**Conclusion:** Combining TFA and MD simulation is efficient for optimizing the reaction conditions, exploring underlying mechanisms, and selecting aptamers in aptamer-target bioassays.

## 1 Introduction

Nucleic acid aptamers, which are artificial ligands of biomolecules, are selected from synthetic random single-stranded oligonucleotide libraries through systematic evolution of ligands by exponential enrichment (SELEX) (Ellington and Szostak, 1990; Tuerk and Gold, 1990; Wu et al., 2021). Aptamers have similar binding properties to antibodies, but they are more stable and easier to prepare and modify than antibodies. Therefore, aptamers are an ideal tool for biomedical molecular detection and recognition (Zhu et al., 2015; Emrani et al., 2016; Li et al., 2019). However, the binding properties of aptamers to their targets are closely related to their structures (Davydova et al., 2020), and the structures of aptamers are sensitive to the conditions of the reaction system. Hence, the analysis of the three-dimensional structures of aptamers and their influencing factors is important for optimizing their binding properties and for selecting preferred aptamers.

Traditionally, the structural analysis of aptamers mainly depends on nuclear magnetic resonance (NMR) (Longhini et al., 2016; Cai et al., 2018) and X-ray crystallography (Ruigrok et al., 2012; Christina Schmidt et al., 2020), but universal application of these methods is not possible due to expensive instruments and complicated procedures. With the rapid development of artificial intelligence technologies, molecular structure prediction based on bioinformatics and molecular dynamics (MD) simulations is increasingly recognized and applied (Douaki et al., 2022; Poolsup et al., 2023), because structural changes in aptamers (before and after binding to their targets) can be rapidly analyzed without damaging the aptamers, the effects of metal ions in the reaction system on the interaction of aptamers and targets can be analyzed, and information on the stability, binding energy, affinity, and molecular mechanism of aptamer-target binding is provided (Hayashi et al., 2014; Hilder and Hodgkiss, 2017; Cui et al., 2020). Through MD simulations, La Penna *et al.* (La Penna and Chelli, 2018) found that Mg^2+^ and Na^+^ could attach to the aptamer surface to enhance the interaction between the amino acid residues of protein targets and the nucleotides of aptamers; thus, Mg^2+^ and Na^+^ facilitated the stability of the binding compounds. Using MD simulations, Vu *et al.* (Vu et al., 2018) identified the binding site of platelet-derived growth factor-B (PDGF-B) with its specific aptamer, and their binding force was mainly the electrostatic force between the positively charged amino acid of the target and the negatively charged phosphate backbone of the aptamer.

In general, it is necessary to select excellent aptamers from the dozens of aptamers generated by SELEX for subsequent applications (Darmostuk et al., 2015; Vu et al., 2018). Specificity and affinity analyses are traditional methods for selecting aptamers, but these methods are labor-intensive and often inflexible when used in complex assay settings. Thermofluorimetric analysis (TFA) is a method that can be used to evaluate aptamer-target binding and distinguish bound and free aptamers in a convenient and rapid manner. This method is based on the melting curve analysis in a polymerase chain reaction (PCR) system (Hu and Easley, 2017), and it can be used to optimize experimental conditions and select the optimal aptamer in complex reaction systems. TFA has been applied to quantify serum platelet-derived growth factor (PDGF), insulin, and prothrombin based on aptamers (Hu et al., 2015; Kim et al., 2015).

Binding between a target and an aptamer is primarily governed by the mutual adaptation of their conformations (the so-called “lock and key” relationship) (Hayashi et al., 2014), and intermolecular non-covalent bonds such as hydrogen bonds, electrostatic interaction, and van der Waals forces act as auxiliary factors in the binding between aptamer and target (Shigdar, 2019; Morozov et al., 2021), while there are also reports of aptamer and target being bound via covalent bonds (Tivon et al., 2021; Tabuchi et al., 2022). Metal ions can impact the tertiary structure of aptamers and their ability to bind to targets through their charge distribution and potential energy (Moccia et al., 2019). Therefore, it is necessary to optimize the metal ion concentrations in binding buffers to identify aptamers with a wider range of adaptability and high specificity. Conventional methods for optimization are generally based on multifactor, multilevel experiments, which are time-consuming and sometimes yield unsatisfactory results (Cheng et al., 2003; Zhou et al., 2015). Thus, a simple, fast, and effective method is needed to optimize the metal ions in the buffers.

The status of aptamer-target interactions can be simply and rapidly observed through TFA, and aptamer structures and their interactions with targets can be visually analyzed through MD simulation. Thus, we speculated that by combining TFA and MD simulations, the effects of buffers and metal ions on aptamer structures and their interactions with targets can be easily analyzed at both experimental and theoretical levels, which can help to select optimal aptamers and optimize experimental conditions. In the present study, the reported aptamer AP273 against alpha-fetoprotein (AFP) (Dong et al., 2015) and our screened aptamer AP-L3-4 against AFP-L3 were used as models for validation, by which we attempted to provide a simple and feasible method for prioritizing aptamers and optimizing experimental conditions.

## 2 Materials and methods

### 2.1 Determination of the optimal aptamer concentration by TFA

Aptamers AP273 (5′-GTG ACG CTC CTA ACG CTG ACT CAG GTG CAG TTC TCG ACT CGG TCT TGA TGT GGG TCC TGT CCG TCC GAA CCA ATC-3′) and AP-L3-4 (5′-ACC GAC CGT GCT GGA CTC TGT CGA AAG GAT ACT GAG TAT TGA GGG GCG TCA GGT GGA AGA GTA TGA GCG AGC GTT GCG-3′) were synthesized (Sangon Biotech (Shanghai) Co., Ltd.) and dissolved in ddH_2_O to prepare a 100 nM storage solution. AP273 is an ssDNA aptamer against AFP selected using CE-SELEX by Wu’s team (Dong et al., 2015); AP-L3-4 is an ssDNA aptamer against AFP-L3 selected using SELEX in our previous work.

The optimal concentration of aptamer AP273 for binding with AFP was determined by analysing the characteristics of the melting curves generated by different concentrations of aptamer reacting with a fixed concentration of AFP. Gradient concentrations of aptamer working solutions (1.25, 2.5, 5.0, 10, 20, 40, and 80 nM) were prepared by adding the typical buffer used in aptamer screening (HEPES-Na 20 mmol/L, NaCl 120 mmol/L, KCl 4 mmol/L, 2 mmol/L MgCl_2_, 1 mmol/L CaCl_2_, pH 7.35). Human recombinant AFP (Nearshore Protein Technology Ltd., Shanghai, China) was dissolved in ddH_2_O to prepare a solution of 1.45 nM (100 ng/mL). EvaGreen (20×) dye (Biotium, United States of America) was diluted to 8× working solution using ddH_2_O (EvaGreen is a nucleic acid fluorescent dye commonly used in real-time PCR (Shoute and Loppnow, 2018).

The aptamer working solution was denatured in a metal bath at 95°C for 3 min and then immediately placed in an ice bath for 3 min. Twenty microliters of the denatured solution was added to PCR tubes, followed by the addition of 5 µL of 8× Evagreen working solution and 5 µL of human recombinant AFP solution. This mixture was incubated for 30 min at room temperature and then placed in a StepOnePlus™ real-time PCR system (Applied Biosystems Inc., United States of America) to measure melting curves (from 4°C to 80°C, with a 0.5°C rise every 10 s and fluorescence detection). These experiments were performed in three duplicate tubes. An equal volume of the buffer solution instead of the aptamer working solution was used as a blank control.

The melt region derivative data (dF/dT) of each tube was exported from the PCR system. The average dF/dT value of the three replicate tubes at each temperature point was calculated and then corrected by subtracting the average dF/dT value of the three blank control tubes. The dF/dT values were normalized (0–100). The melting curves were plotted with the temperature as the *X*-axis and the corrected or normalized dF/dT values as the *Y*-axis, and the optimal concentration of the aptamer was determined based on the melting temperature (Tm), peak height and peak area.

### 2.2 Determination of the optimal buffer system by TFA

To determine the optimal buffer for the experiment, the interaction of aptamer AP273 (at the optimal concentration determined above) and AFP (1.45 nM) was performed in three buffers (20 mM HEPES, 10 mM PBS and 20 mM Tris-HCl, pH = 7.35–7.45) with constant metal ion concentrations (Na^+^ 140 mM, Mg^2+^ 2 mM, K^+^ 4 mM, and Ca^2+^ 1 mM), and then the melting curves were measured via TFA. The experimental procedure and interpretation of the results were the same as described above.

### 2.3 Determination of optimal metal ion strength by TFA

The optimal metal ion strength for the experiment was also determined via TFA. The concentration points of metal ions were set according to the results reported in the literature as follows (Duan et al., 2012; Kang et al., 2015; Wu et al., 2017; Chen et al., 2020a): 1, 2 and 5 mM for Mg^2+^; 100, 120 and 140 mM for Na^+^; 2, 4 and 5 mM for K^+^; and 1, 2 and 2.5 mM for Ca^2+^. A 4-factor, 3-level orthogonal test (with a fixed random seed number of 300) was designed to determine the optimal concentration of metal ions. Based on the orthogonal design, nine buffers with different concentrations of metal ions were prepared using the optimal buffer system determined in the previous step (Table 1). The concentrations of aptamers and AFP and the experimental procedures were the same as described above. In subsequent MD analysis, the buffers with the optimal metal ion combination were selected according to the Tm value, peak height and peak area.

**TABLE 1:** Concentrations of metal ions in buffers based on orthogonal design.

Buffer	Metal ion concentration (mM)			
	Mg^2+^	Na^+^	K^+^	Ca^2+^
1	1	120	5	2.5
2	5	100	4	2.5
3	2	140	2	2.5
4	5	140	5	1
5	2	100	5	2
6	1	100	2	1
7	1	140	4	2
8	5	120	2	2
9	2	120	4	1

### 2.4 Structure prediction, docking and MD simulations of the aptamers to AFP

Structure prediction and docking of two aptamers and AFP were performed by the following software: 1) The secondary structure of the aptamers was predicted using the online software Mfold (http://www.unafold.org/hybrid2.php) (Zuker, 2003). Aptamer structures with the minimum energy (the lowest ΔG value) were selected. The ct. output formats (dot-bracket notation) were used for the construction of the aptamer 3D structures (Garcia-Recio et al., 2016; Heiat et al., 2016; Subki et al., 2020). 2) The tertiary structure of aptamers was predicted by using online RNA Composer software on the basis of the secondary structure of aptamers (https://rnacomposer.cs.put.poznan.pl) (Popenda et al., 2012; Antczak et al., 2016). 3) The nucleotide change for the aptamer was performed by using DS (Biovia Discovery Studio) software (the uracil was changed to thymine, and the ribose backbone was changed to deoxyribose) (Chang et al., 2020). 4) All-atom (AA) energy minimization simulations of the aptamer were performed in a vacuum by using software NAnoscale Molecular Dynamics (NAMD) (Phillips et al., 2005) with the Visual Molecular Dynamics (VMD) (Humphrey et al., 1996). 5) The 3D model of human AFP was obtained from either the AlphaFoldDB website (https://alphafold.ebi.ac.uk/) using code number AF-J3KMX3-F or the PDBe-KB website (https://www.ebi.ac.uk/pdbe/pdbe-kb/proteins/P02771) with the PDB code 7YIM (UniProt et al., 2023). 6) Molecular docking of the 3D structure of the aptamer and AFP was performed online using ZDOCK 3.0.2 (https://zdock.umassmed.edu/) (Mintseris et al., 2007; Pierce et al., 2011; Pierce et al., 2014). 7) The model with the highest ZDOCK score (Wang et al., 2019) was selected as the initial structure for MD simulations, using Amber 18 software (Lee et al., 2020), in which the aptamer was assigned OL15 nucleic acid force fields (Zgarbova et al., 2015) and the target molecule was assigned ff14SB protein force fields (Tian et al., 2020). The reaction system solvent cassette (Steinberg et al., 2019) was set up according to the metal concentrations screened by the TFA experiment. 8) Docking, MD simulation results, hydrogen bonding, and binding sites were visualized using PyMOL software (The PyMOL Molecular Graphics System, Version 2.0 Schrödinger, LLC) (Lill and Danielson, 2011). The optimal buffer system was determined based on comprehensive analyses of the simulations.

### 2.5 Binding characteristics of aptamers to AFP under different ionic conditions

With optimal buffering conditions, TFA was performed for both fixed and gradient concentrations of aptamers to verify their binding characteristics to AFP. The experimental procedure and data processing were the same as above, and the binding characteristics of aptamers to AFP under various buffer systems were analyzed according to the Tm value, peak height and peak area.

### 2.6 Comparisons between different aptamers

In order to verify whether the combination of TFA and MD simulations can distinguish different aptamers and identify the optimal aptamer, the same experiments were performed with the aptamer AP-L3-4. Differences in the binding of aptamers AP273 and AP-L3-4 to AFP were compared, including the binding site, the distance and frequency of hydrogen bonds, binding energy, and binding characteristics for the gradient concentrations of AFP.

## 3 Results

### 3.1 Optimal concentration of aptamers determined by TFA

**FIGURE 1:**
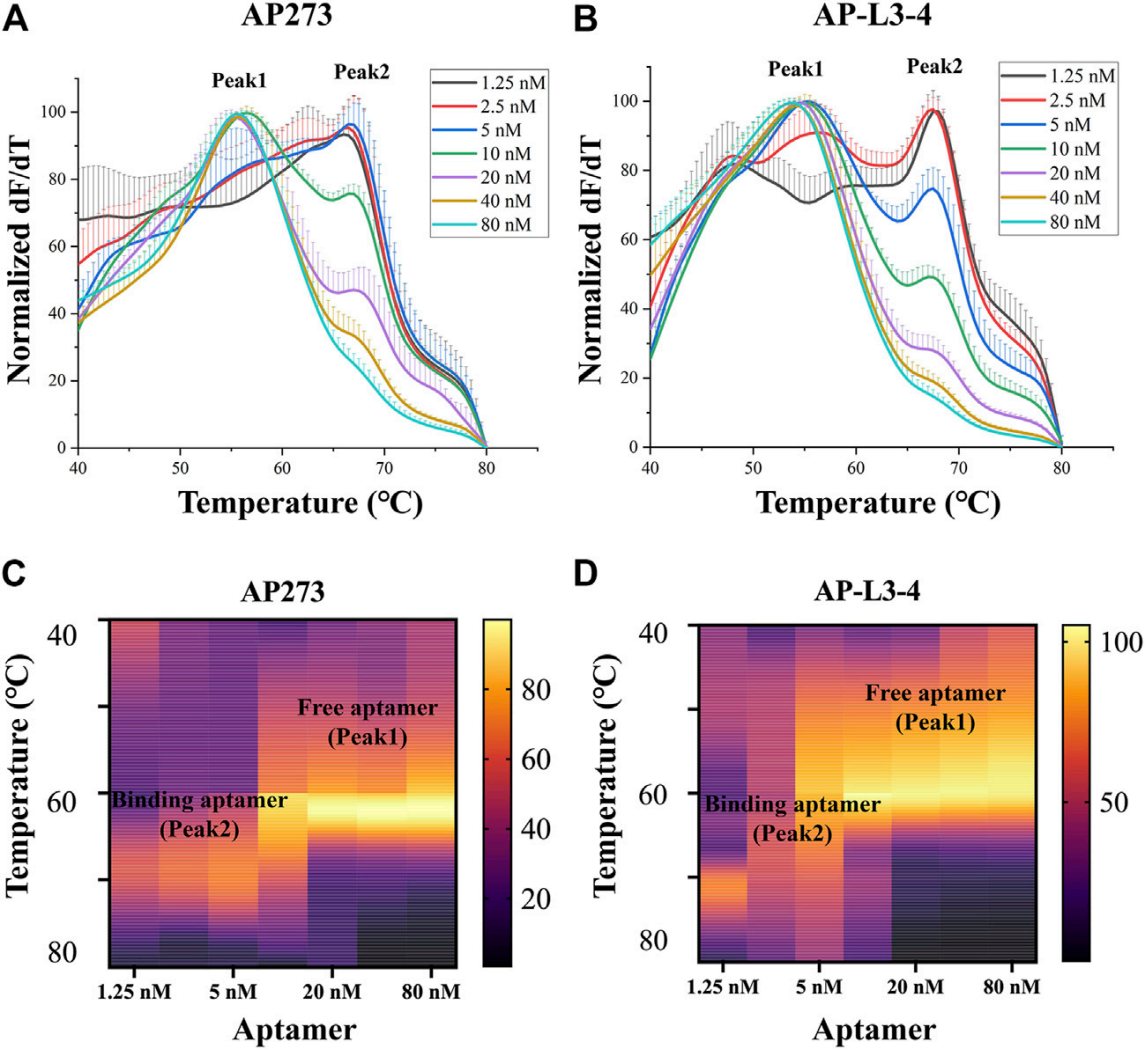
Thermofluorimetric analyses of the gradient concentrations of aptamers binding to AFP. **(A)** Melting curves of AP273 at 1.25–80 nM. **(B)** Melting curves of AP-L3-4 at 1.25–80 nM. **(C)** Heatmap of the area under the peaks of AP273 at 1.25–80 nM. **(D)** Heatmap of the area under the peaks of AP-L3-4 at 1.25–80 nM.

The melt curves of normalized dF/dT value and the heatmaps of peak area for gradient concentrations of aptamers AP273 and AP-L3-4 binding to AFP are shown in Figure 1. The free aptamer peak (peak 1) of AP273 appeared at 2.5 nM and then rose with increasing concentration and peaked at 10 nM, while the bound aptamer peak (peak 2) almost peaked at 2.5 nM and then gradually decreased from 10 nM (Figure 1A). A similar trend was found in aptamer AP-L3-4, but the free aptamer peak topped out and the bound aptamer peak started to decrease earlier (both at 5 nM) (Figure 1B). The heatmaps of the area under the peaks showed that the bound aptamer peak of AP273 was stronger than that of AP-L3-4 at the same aptamer concentration, while the free aptamer peak was the opposite (Figures 1C, D). These findings suggest that TFA can exhibit concentration-dependent changes in aptamer-target interactions and reflect differences between aptamers.

**FIGURE 2:**
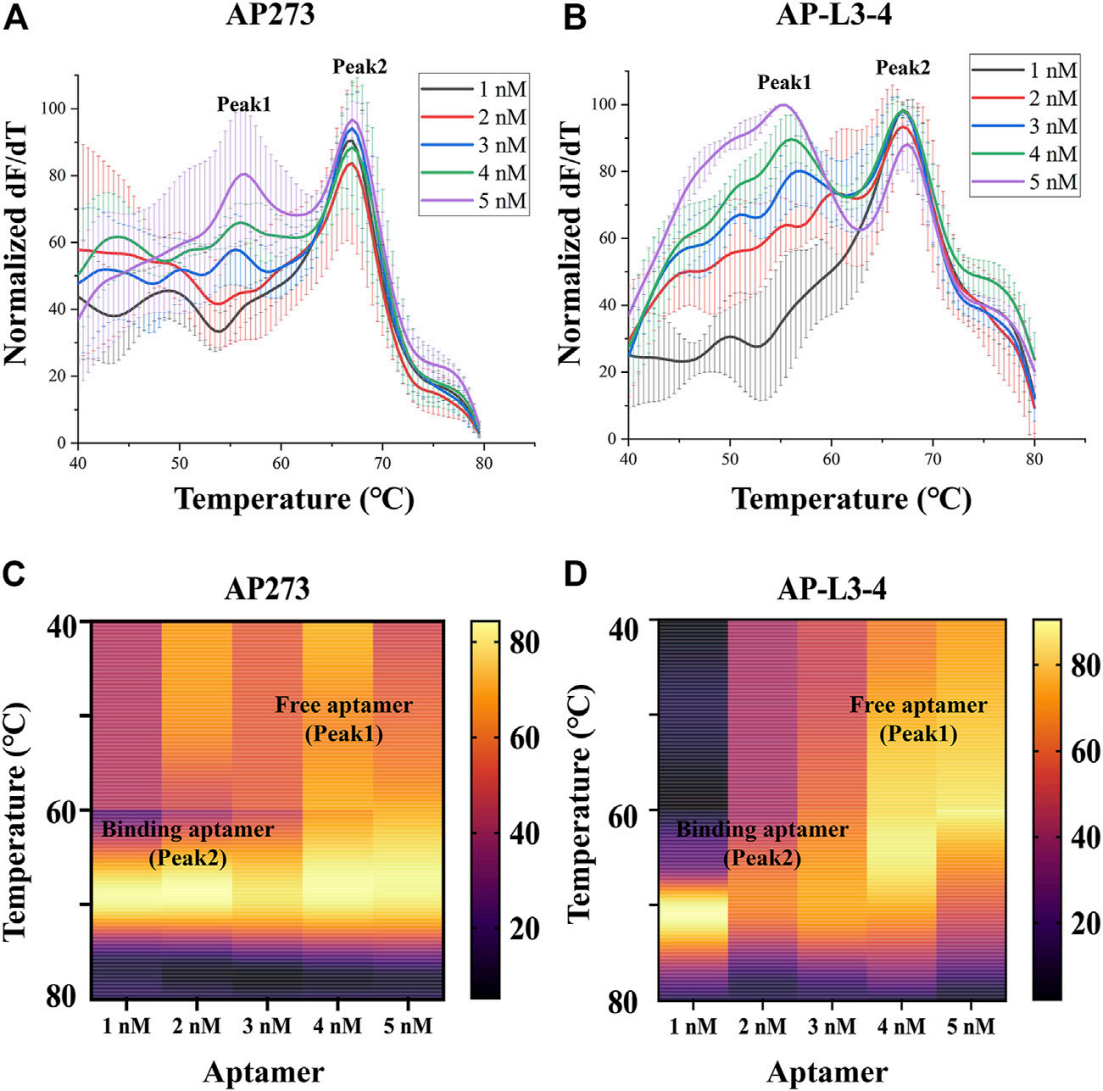
Thermofluorimetric analyses of the refined gradient concentrations of aptamers binding to AFP. **(A)** Melting curves of AP273 at 1–5 nM. **(B)** Melting curves of AP-L3-4 at 1–5 nM. **(C)** Heatmap of the area under the peaks of AP273 at 1–5 nM. **(D)** Heatmap of the area under the peaks of AP-L3-4 at 1–5 nM.

The results of refinement experiments for both aptamers at concentrations of 1–5 nM are shown in Figure 2. For AP273, the bound aptamer peak (peak 2) appeared at the lowest concentration (1 nM) and was maintained to the maximum concentration (5 nM), and its free aptamer peak (peak 1) appeared at 3 nM and continuously rose (Figure 2A). A similar trend was observed in AP-L3-4, although the free aptamer peak was more pronounced (Figure 2B). The heatmaps of the area under peak showed that AP273 had stronger bound aptamer peaks and weaker free aptamer peaks compared with AP-L3-4 (Figures 2C, D). Considering the need to detect different concentrations of AFP, we selected 4 nM as the optimal concentration for subsequent experiments.

### 3.2 Optimal buffer system and ion concentration for aptamer-target interactions determined by TFA

**FIGURE 3:**
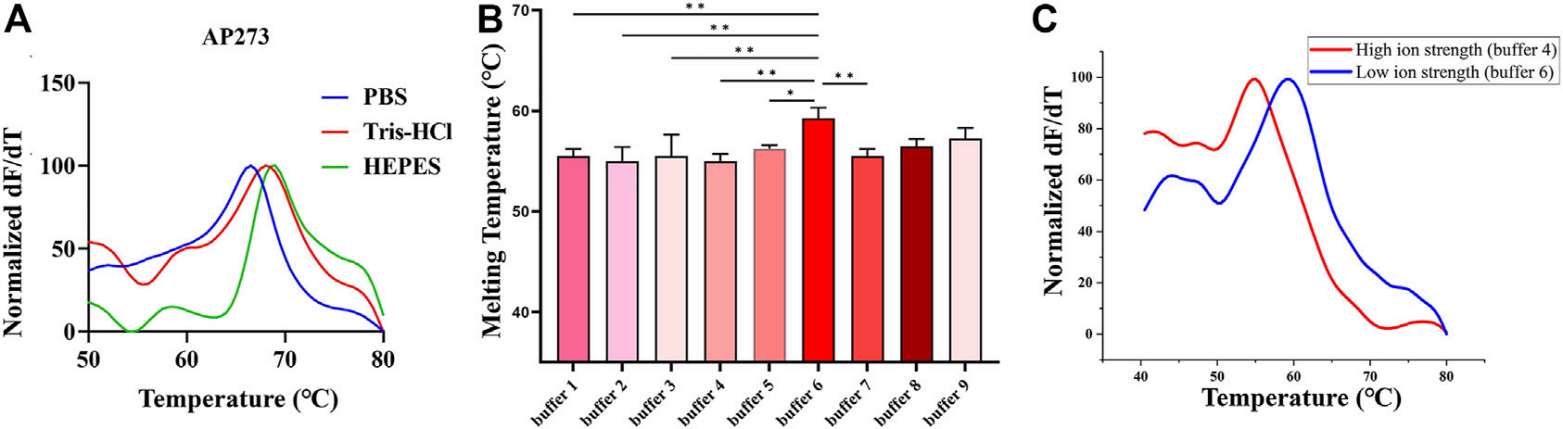
Thermofluorimetric analyses of AP273 binding to AFP in different buffer and ionic strength conditions. **(A)** Melting curves in different buffer systems. **(B)** The melting temperatures in HEPES buffer system with various metal ion concentrations. *p < 0.05, **p < 0.01. **(C)** Melting curves of high and low ionic strength buffer solutions.

The melting curves of AP273 (4 nM) binding to AFP (1.45 nM) in three buffer systems (HEPES, PBS, Tris-HCl) showed different Tm values (Figure 3A), of which the HEPES buffer system had the largest Tm value and therefore was selected as the optimal buffer system for subsequent experiments. The Tm values of AP273 binding to AFP also varied in HEPES buffer solutions (buffer 1–9) with various metal ion concentrations (according to the orthogonal design) (Figure 3B), with the lowest Tm value in buffer 4 and the highest Tm value in buffer 6. The Tm value was higher in the buffer solution with the lowest ionic strength (buffer 6) than in the buffer solution with the highest ionic strength (buffer 4) (Figure 3C). Higher Tm values indicate more stable binding between aptamers and targets, and thus buffers 5, 6, and 9, which had higher Tm values were selected as buffer solutions for the next molecular dynamics analysis, and buffer 7 with the same concentration of Mg^2+^ ions, was selected as the control buffer solution.

### 3.3 Secondary structure prediction of AP273 and its free energy

**FIGURE 4:**
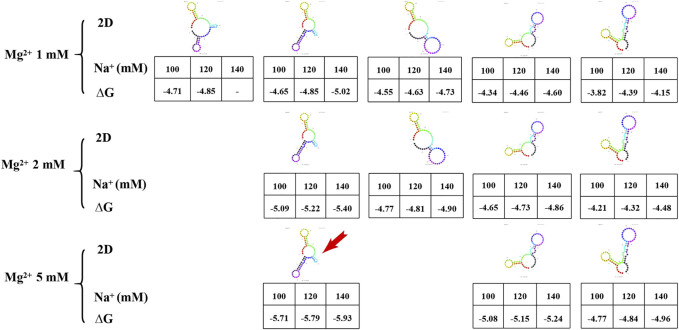
The secondary structure and free energy (∆G) of AP273 predicted at different ion concentrations. The colour figures: secondary structures of AP273. Table: Na^+^ concentration in the first row, ∆G value in the second row. Red arrow: the secondary structure selected for the prediction of tertiary structure.

Prediction of the secondary structure is required before the tertiary structure of an aptamer can be determined. In the prediction of secondary structures of aptamer AP273, it was found that the amount and minimum free energy of secondary structure varied with the concentrations of Na^+^ and Mg^2+^ ions (Figure 4). Increased concentrations of Mg^2+^ ions led to fewer predicted secondary structures. The concentration of Na^+^ ions did not affect the amount of secondary structure, but it impacted the minimum free energy of the secondary structure. Under the same concentration of Mg^2+^ ions, higher concentrations of Na^+^ ions led to lower free energy values. The structure with the lowest value of ∆G (the most stable secondary structure) was selected for subsequent prediction of the tertiary structure.

### 3.4 Molecular dynamics simulation of the interaction between AP273 and AFP

**FIGURE 5:**
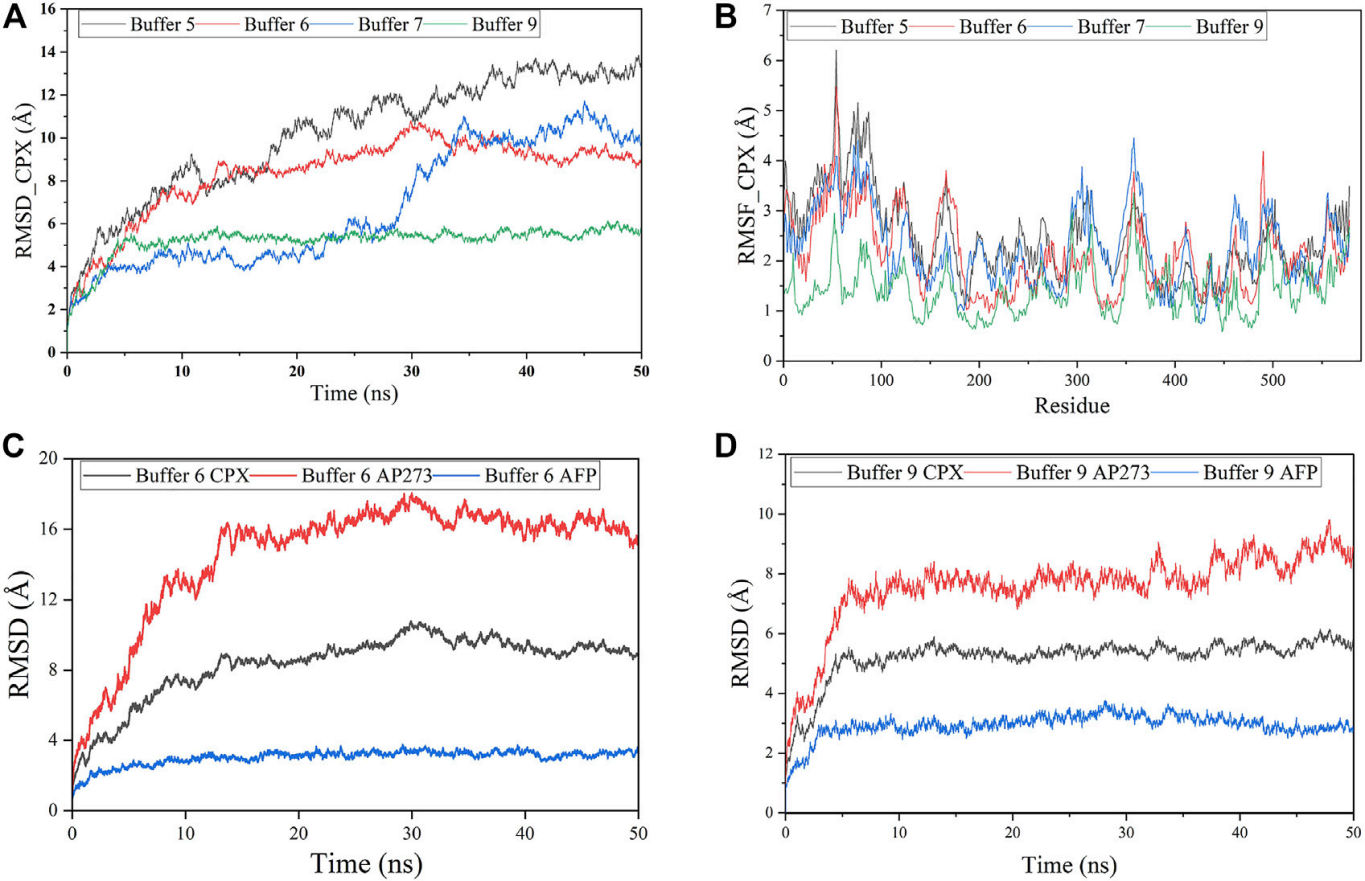
Molecular dynamics simulations of aptamer AP273 binding to AFP. **(A)** The root means square deviation (RMSD) of the complex of aptamer AP273 and AFP (CPX) in various buffer solutions. **(B)** The root mean square fluctuation (RMSF) of residues during MD simulations in various buffer solutions. **(C)** and **(D)** The RMSDs of CPX, AFP, and AP273 in buffer 6 and buffer 9.

The docking models of AP273 and AFP were then subjected to MD simulations in different buffer solutions (Figure 5). The results of root mean square deviation (RMSD) (Figure 5A) and root mean square fluctuation (RMSF) (Figure 5B) showed that the stability of the complex of AP273 and AFP was the highest in buffer 9 and higher than that in buffer 6. The comparisons of AP273, AFP and their complex between buffer 6 and buffer 9 (Figures 5C, D) exhibited that AFP had the highest stability and AP273 had the lowest stability, and that the AP273-AFP complex was more stable in buffer 9 than in buffer 6. These results suggest that the metal ions in buffer systems can affect the stability of aptamer-target complexes.

The free energy of AP273 binding to AFP in various buffer solutions was calculated by the **MM/GBSA** method (Rastelli et al., 2010; Hou et al., 2011; Genheden and Ryde, 2015; Chen et al., 2020b) and is shown in Table 2. As shown in Table 2, buffer 6 exhibited the largest binding energy but not the best stability (Figure 5A), which is not consistent with the results of TFA experiments, indicating that the binding force and stability of aptamer-target are influenced by other complex environmental factors, such as temperature and pH, etc.

**TABLE 2:** Binding free energies of AP273 and AFP predicted by MM/GBSA (kcal/mol).

	Buffer 5	Buffer 6	Buffer 7	Buffer 9
Δ*E*_vdw_	−243.4	−213.4	−205.17	−223.32
Δ*E*_elec_	8,949.21	8,634.37	9,121.31	10,193.54
ΔG_GB_	−8,763.83	−8,517.34	−8,989.8	−10,041.84
ΔG_SA_	−30.64	−27.03	−26.71	−28.68
ΔG_bind_	−88.67	−123.4	−100.37	−100.31

AFP, alpha-fetoprotein; MM/GBSA, molecular mechanics generalized born surface area; Δ*E*_vdw_, van der Waals energy; Δ*E*_elec_, electrostatic energy; ΔG_GB_, electrostatic contribution to solvation; ΔG_SA_, nonpolar contribution to solvation; ΔG_bind_, binding free energy.

**FIGURE 6:**
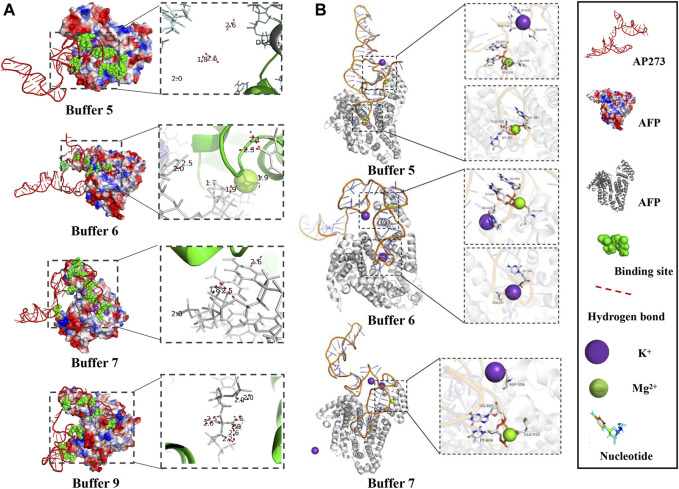
Visualization of MD simulation results in various buffer solutions. **(A)**. Binding sites and the hydrogen bonds between AP273 and AFP in four buffer solutions. **(B)**. The interaction of K^+^ and Mg^2+^ ions with the complex of aptamer AP273 and AFP in MD simulations.

The visualization of the MD simulation results provided qualitative binding information between AP273 and AFP in different buffer solutions (Figure 6), which indicated that the binding sites and hydrogen bonds were different under various buffer solutions and that metal ions were involved in the binding.

**FIGURE 7:**
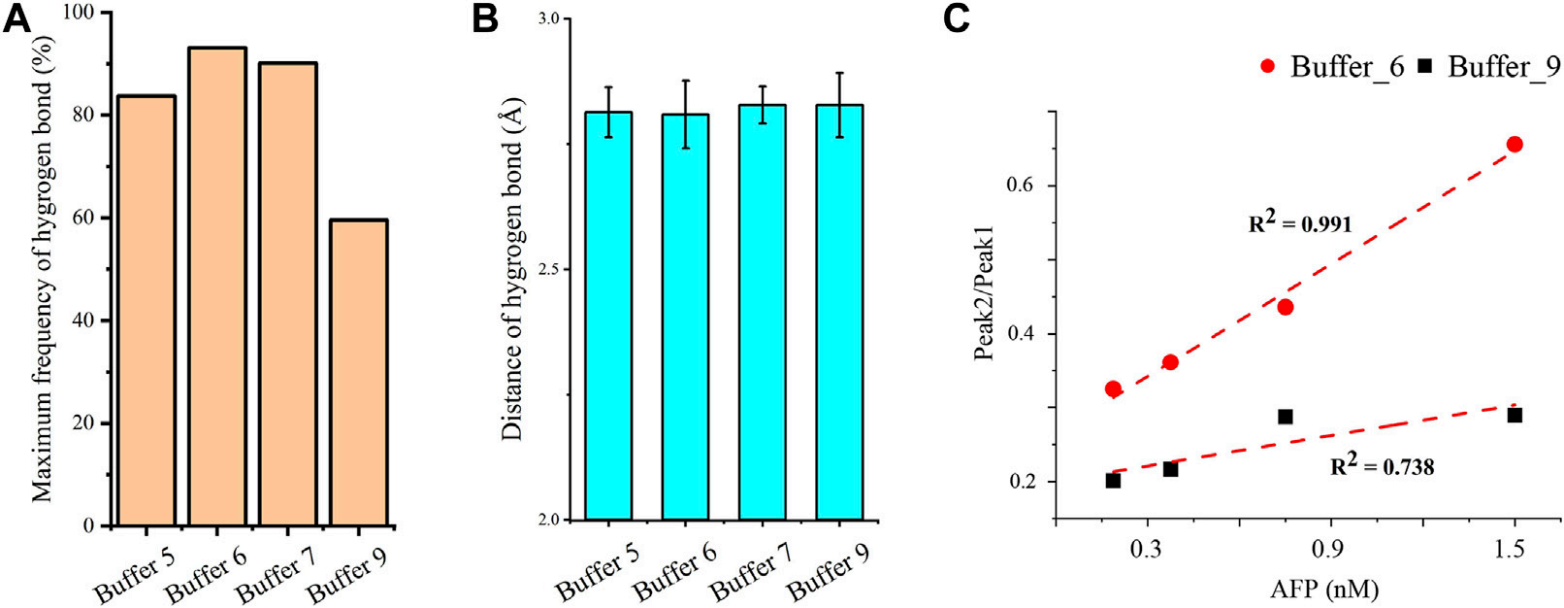
Quantitative analyses of hydrogen bonds and linear correlations in the interaction between AP273 and AFP. **(A)** and **(B)** The maximum frequency and distance of hydrogen bonds in the four buffer solutions. **(C)** The linear correlations between AP273 and AFP in buffer 6 and buffer 9.

The hydrogen bonds in AP273 binding to AFP under four buffer solutions were further quantified (Figures 7A, B). The results showed that the frequency of hydrogen bonds was the lowest in buffer 9 and the highest in buffer 6, and that the distance of hydrogen bonds was similar in the four buffer solutions, suggesting that the hydrogen bond frequency rather than hydrogen bond distance plays an important role in the binding between AP273 and AFP. TFA experiments were conducted under buffer 6 and buffer 9, and results showed that buffer 6 was more favorable than buffer 9 for AP273 to bind to AFP in terms of bound aptamer ratio and linear correlation (Figure 7C).

### 3.5 Comparison between aptamers AP273 and AP-L3-4

**FIGURE 8:**
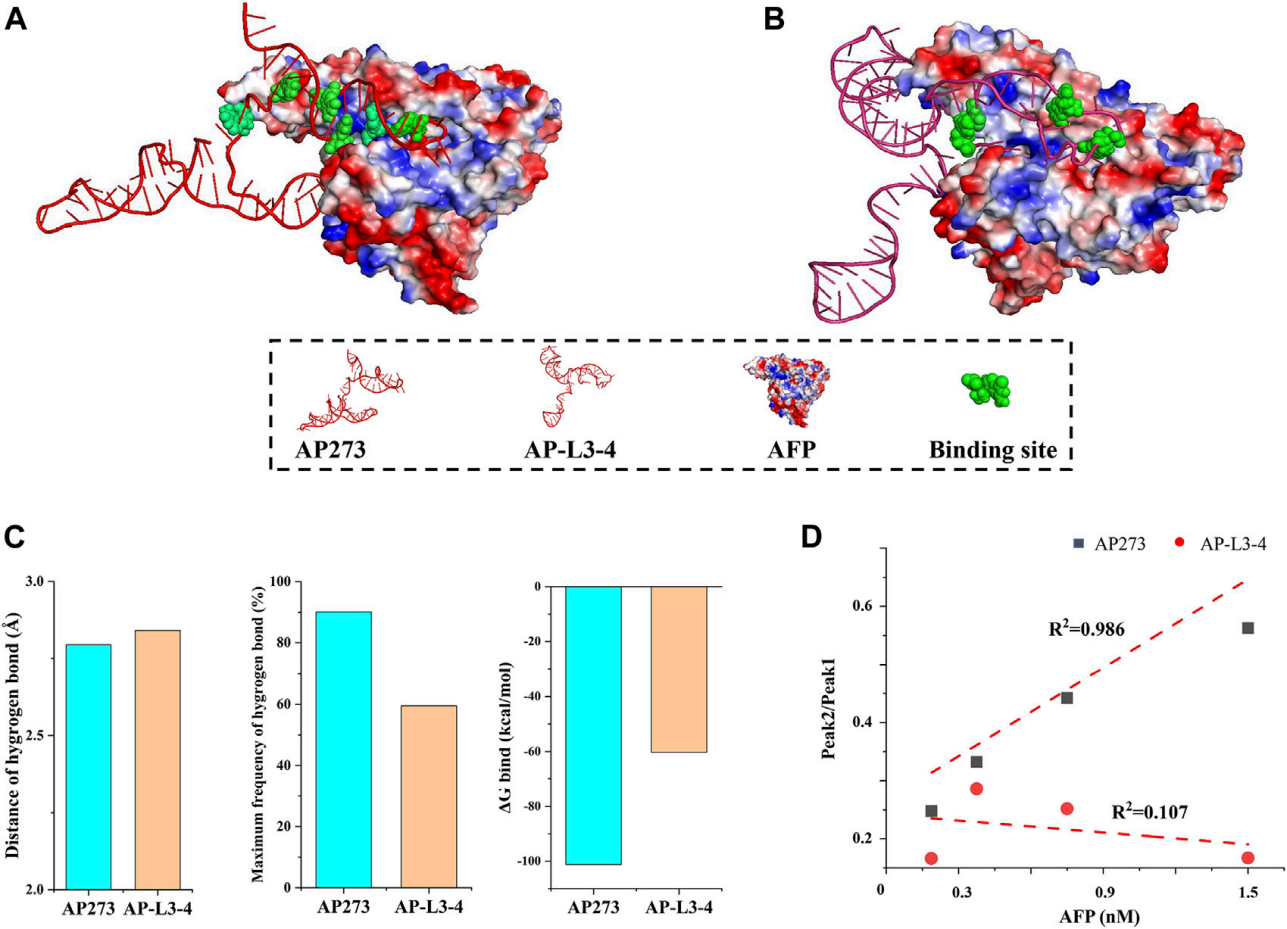
Comparison of aptamers AP273 and AP-L3-4 binding to AFP in molecular dynamics simulation and thermofluorimetric analysis. **(A)** and **(B)** Docking models of AP273 and AP-L3-4 with AFP and their binding sites (green); **(C)** Distances and frequencies of hydrogen bonds and binding energies of AP273 and AP-L3-4 bound to AFP; **(D)** The linear correlations of AP273 and AP-L3-4 bound to AFP in thermofluorimetric analysis.

Comparative studies were performed between aptamers AP273 and AP-L3-4 to further verify whether the combination of TFA and MD simulation could select the aptamer with better binding properties. Compared with AP-L3-4, AP273 binding to AFP in MD simulations had more binding sites (Figures 8A, B), a shorter hydrogen bond distance, a higher hydrogen bond frequency, and less free energy (Figure 8C). TFA results showed better linear correlation of AP273 than AP-L3-4 for detecting gradient concentrations for AFP (Figure 8D). These findings demonstrate that the binding property of AP273 to AFP is superior to that of AP-L3-4, suggesting the theoretical and experimental feasibility of combining TFA and MD simulation for preferential aptamer selection.

## 4 Discussion

TFA analyzes the binding of aptamers to their target molecules using melting curves. The free aptamer shows a smaller Tm value than that of the bound aptamer, so two peaks, peak 1 (the free aptamer peak) and peak 2 (the bound aptamer peak), can be observed on the melting curve of TFA (Kim et al., 2015; Mahmoud et al., 2019). In the present study, distinct peak 1 and peak 2 appeared on the melting curves after aptamers AP273 or AP-L3-4 were reacted with AFP, and the Tm value, peak height and peak area were different under various experimental conditions. Using these indicators, the optimal experimental system and the best aptamer can be determined, making TFA useful to optimize the aptamer-target reaction system and to select preferred aptamers. The Tm value of a bound aptamer peak is positively correlated with the stability of aptamer-target binding. In the present study, the Tm values for peak 2 differed across the three buffer systems, with the highest value in the HEPES buffer system, suggesting that the binding of AP273 to AFP is more stable in the HEPES buffer system compared to the other two buffer systems. This may be because HEPES is not prone to form complexes with metal ions in the buffer system and thus exhibits a better buffering effect (Ruzza et al., 2021). Using HEPES as the buffer salt, nine buffer solutions with various metal ion concentrations determined by orthogonal design were formulated for TFA experiments to analyze the effects of metal ions on the binding of AP273 to AFP. The Tm values of peak 2 were maximum in buffer 6 (with the lowest ionic strength) and minimum in buffer 4 (with the highest ionic strength), indicating that the high ionic strength is unfavorable for the binding of AP273 to AFP molecules. This is consistent with previous reports that a buffer solution with relatively low ionic strength is more appropriate for TFA experiments (Hayashi et al., 2014) and that immobilization of aptamers under low ionic strength conditions rather than conventional high ionic strength buffers can greatly improve the performance of the E-AB sensor (Liu et al., 2021).

The metal ions of the buffer system are important in aptamer-target binding. In the secondary structure prediction, we found that the number of secondary structures of AP273 varied with the Mg^2+^ and Na^+^ concentrations and that the Mg^2+^ concentration was proportional to the free energy (absolute value of ΔG) of the aptamer secondary structure. In MD simulations, we found that Mg^2+^ and K^+^ contributed to hydrogen bonds at the binding site of the aptamer to the target molecule (Figure 6B). Metal ions are mainly embedded in the helical grooves of aptamer tertiary structures and affect the binding properties of aptamers to the target molecules in different ways (McCluskey et al., 2019). Metal ions can neutralize the negative charge of the phosphate groups of aptamers to affect the amount of charge in aptamer structures, hence affecting the structure of aptamers; in this context, Mg^2+^ affects the flexibility of aptamers and the stability of aptamer-target compounds (Zhang and Yadavalli, 2010). The aptamer structure is unstable in the binding buffer without Mg^2+^ (Zhao et al., 2020), and Mg^2+^ is important for stabilizing the two- and three-dimensional structures of aptamers (Huang et al., 2013). In addition to Mg^2+^, the Na^+^, K^+^ and pH value of a buffer system also have an effect on the binding of aptamers to targets, and the optimization of them can significantly improve the efficiency of aptamer sensors in detecting targets (Hianik et al., 2007; Belleperche and DeRosa, 2018) and significantly increase the sensitivity of aptamer sensors (Yang et al., 2022). The binding of thrombin to its aptamer also depends on pH and electrolytes (Hianik et al., 2007). The pH value may affect the ssDNA conformational changes and the electron transfer between the target protein and the aptamer, which is related to the maintenance of the three-dimensional conformation of the aptamer; and an increase in Na^+^ concentration leads to a weaker binding of thrombin to the aptamer, possibly due to the shielding effect of Na^+^ ions on the target. In addition, many studies have confirmed that K^+^ ions can stabilize the structure of the aptamer, particularly for G-quadruplexes (Santos et al., 2022).

The stability and affinity of aptamer-target complexes involve intermolecular covalent bonds, hydrogen bonds, electrostatic interactions and van der Waals forces, in which hydrogen bonds are one of the strongest non-covalent interactions, and the number and distance of hydrogen bonds contribute to the affinity between aptamers and target molecules (Sabri et al., 2019). In the MD simulations, we found that more hydrogen bonds were formed in the binding of AP273 to AFP in buffer 6 than in buffer 9, indicating a stronger binding affinity between the two molecules in buffer 6. However, the most stable binding of AP273 to AFP was found in buffer 9 rather than buffer 6, where the hydrogen bonds were less frequent but the aptamer-target binding sites were more numerous and dispersed. This may suggest the stability is more related to the binding sites of the aptamer and the target, while the affinity is more related to the hydrogen bonds between the aptamer and the target.

In the comparative study of binding properties between aptamer AP273 and aptamer AP-L3-4, we found that the binding properties of AP273 were superior to those of AP-L3-4 in both TFA and MD simulations, suggesting that the combination of TFA and MD simulation can identify aptamers that exhibit better binding characteristics among homologous aptamers.

Additionally, we found that the stability of AP273 binding to AFP was inconsistent between TFA and MD simulations. In TFA experiments, the most stability was found in buffer 6, followed by buffer 9, buffer 5 and buffer 7, while in MD simulations, the most stability was observed in buffer 9, followed by buffer 6, buffer 7 and buffer 5. This may be due to subtle differences in experimental conditions between MD simulations and TFA. The MD simulations were performed under conditions of constant temperature (25°C), neutral reaction environment, atmospheric pressure, aqueous metal ion solution, and no buffer salt, while the TFA were performed under the HEPES buffer system and constantly increasing temperature. Furthermore, it is important to keep in mind the limitations of molecular simulation methods. The results of MD are microscopic simulations and predictions from large data sets; the prediction of aptamer 3D structure based only on the sequence is still a very unreliable task, which has by no means achieved the maturity of protein structure prediction. There is no guarantee that the prediction of an aptamer structure is close to the correct fold. Certainly, an all-atom energy minimization is not sufficient to assess the stability of the predicted aptamer structure, let alone when done in a vacuum.

## 5 Conclusion

In this study, the binding of aptamer AP273 to its target AFP was analyzed by TFA and underlying molecular mechanisms were analyzed through MD simulations. On the melting curve of TFA, the free and bound AP273 peaks were clearly visible, and the binding status of AP273 with AFP could be determined based on the peak-related indicators (Tm value, height or area of the peaks, and the ratio of the two peaks). These indicators varied with changes in aptamer-target ratio, buffer system and metal ionic strength, which can be useful for optimizing experimental conditions. The analysis of MD simulation showed that the affinity and stability of AP273 binding to AFP varied at different metal ionic strengths, and the underlying mechanisms were related to the difference in the hydrogen bond frequency and binding sites. In the comparative study of AP273 and the control aptamer AP-L3-4, the two aptamers were different in the peak-related indicators and in hydrogen bonds and binding free energies, which are useful for the preferential selection of aptamers. Although the stability of AP273 binding to AFP was inconsistent between TFA and MD simulation, the combination of the two methods provided dual validation that was more intuitive than mere experimental results and more convincing than mere theoretical analysis, and it will facilitate the translational application of aptamers in bioassays.

## Data Availability

Publicly available datasets were analyzed in this study. This data can be found here: https://alphafold.ebi.ac.uk/; https://www.ebi.ac.uk/pdbe/pdbe-kb/proteins/P02771.
